# Timing of Maximal Weight Reduction Following Bariatric Surgery: A Study in Chinese Patients

**DOI:** 10.3389/fendo.2020.00615

**Published:** 2020-09-15

**Authors:** Ting Xu, Chen Wang, Hongwei Zhang, Xiaodong Han, Weijie Liu, Junfeng Han, Haoyong Yu, Jin Chen, Pin Zhang, Jianzhong Di

**Affiliations:** ^1^Department of Bariatric & Metabolic Surgery, Shanghai Jiao Tong University Affiliated Sixth People's Hospital, Shanghai, China; ^2^Department of Endocrinology and Metabolism, Shanghai Jiao Tong University Affiliated Sixth People's Hospital, Shanghai, China; ^3^Department of Computer Science, Institute for Biomedical Informatics, University of Kentucky, Lexington, KY, United States

**Keywords:** bariatric surgery, Chinese patients, weight reduction, trend, follow up

## Abstract

**Introduction:** Bariatric surgery is a well-received treatment for obesity with maximal weight loss at 12–36 months postoperatively. We investigated the effect of early bariatric surgery on weight reduction of Chinese patients in accordance with their preoperation characteristics.

**Materials and Methods:** Altogether, 409 patients with obesity from a prospective cohort in a single bariatric center were enrolled retrospectively and evaluated for up to 4 years. Measurements obtained included surgery type, duration of diabetic condition, besides the usual body mass index data tuple. Weight reduction was expressed as percent total weight loss (%TWL) and percent excess weight loss (%EWL).

**Results:** RYGB or SG were performed laparoscopically without mortality or complications. BMI generally plateaued at 12 months, having decreased at a mean of 8.78 kg/m^2^. Successful weight loss of >25% TWL was achieved by 35.16, 49.03, 39.22, 27.74, 20.83% of patients at 6, 12, 24, 36, and 48 months after surgery. Overall, 52.91% of our patients had lost 100% of their excess weight at 12 months, although there was a rather wide range among individuals. Similar variability was revealed in women of child-bearing age.

**Conclusion:** Chinese patients undergoing bariatric surgery tend to achieve maximal weight loss and stabilization between 12 and 24 months postoperatively, instead of at >2 years. The finding of the shorter stabilization interval has importance to earlier intervention of weight loss related conditions and women's conception planning.

## Introduction

Obesity is a chronic health condition that is becoming a global issue. The World Health Organization stated in 2016 that more than 1.9 billion adults were overweight, and more than 650 million were obese. Besides lifestyle intervention and medication, bariatric surgery has been proved to be a safe, effective, and durable procedure for weight loss among morbidly obese patients (1). In addition, a number of comorbidities including essential hypertension, type 2 diabetes mellitus, hyperlipidemia, bronchial asthma, obstructive sleep apnea, and osteoarthritis can be ameliorated or even resolved following bariatric surgery. In China, the number of bariatric operations conducted has increased from around 4,000 as of the previous 5 year period to more than 10,000 during the past 5 years (2).

In western countries, research evidences support the consensus that postoperative body weight decreases to a trough at 12–36 months after bariatric surgery (3–5). However, there is currently no published investigation on standardized weight loss after bariatric surgery in Asian settings, except a few studies in Japan and Singapore showed that the average percent total weight loss (%TWL) following bariatric surgery was 20–25% within the first 3 years (6, 7). On account of differences in the baseline body height and weight, and body composition, it is not completely grounded to interpret the weight loss on the Asian communities according to westerner physical standards.

The degree of weight loss and the time when it most likely occurs after bariatric surgery has been insufficiently addressed in China. In addition, we recognized in our patients that women who suffer from obesity-related infertility are more likely to have unplanned pregnancies within the first year following bariatric procedure. These altogether necessitate the establishment of the timing of maximal weight reduction during preoperative counseling. Patients who gain a realistic expectation of weight loss tend to stick to lifestyle changes and better cooperate with medical intervention, leading to uneventful recovery and satisfactory (8). To fulfill the need, we examined the up to 4 years' weight loss responses in Chinese patients following bariatric surgery according to their body mass index (BMI), age, sex, surgery type, and duration of type 2 diabetes mellitus (T2DM).

## Methods

We retrospectively reviewed the medical records of 409 patients who had undergone RYGB between February 2011 and August 2018. The Human Research Review Board of our institution approved the study and all patients provided their written informed consent. The study was conducted in accordance with the principles of the Declaration of Helsinki. Our previous studies focused on the improved renal, respiratory, and reproductive function after surgery (9–13). The present study includes bariatric surgery patients who underwent at least one follow-up evaluation during the first year after their surgery. Exclusion criteria included missing preoperative BMI record, unknown surgery type, death, and/or the presence of surgical complications.

Altogether, 409 patients were enrolled in the study (252 women, representing 61.6%). Among them, 227 underwent Roux-en-Y gastric bypass (RYGB) (55.5%), and the rest underwent sleeve gastrectomy (SG). Data were collected concerning the patients' demographic data, including their age and sex, T2DM diagnosis, type of surgery, initial BMI, and postoperative weight loss for up to 4 years (Table 1). Postoperative weight loss was expressed using the postoperative BMI, change in BMI, %TWL and %EWL.

**Table 1:** Baseline Characteristics.

**Parameter (*n* = 409)**	**Value**
BMI, kg/m^2^, Mean ± SD (range)	34.37 ± 6.25 (25.5,67.5)
BMI group, *n* (%)	
25–27.5 kg/m^2^	22 (5.4%)
27.5–32.5 kg/m^2^	177 (43.3%)
32.5–37.5 kg/m^2^	104 (25.4%)
37.5 kg/m^2^ and above	106 (25.9%)
Male sex, *n* (%)	157 (38.4%)
**T2DM duration**, ***n*** **(%)**	
Non-diabetic	90 (22.0%)
0–5 years	168 (41.1%)
5–10 years	91 (22.2%)
10–15 years	43 (10.5%)
above 15 years	17 (4.2%)
Age, yrs, Mean ± SD	42.43 ± 13.27
**Age**, ***n*** **(%)**	
18–45	235 (57.5%)
45–60	118 (28.9%)
60 and above	56 (13.7%)
RYGB surgical type, *n* (%)	227 (55.5%)

*BMI, body mass index; T2DM, type 2 diabetes mellitus; RYGB, Roux-en-Y gastric bypass*.

### Statistical Analyses

IBM SPSS Statistics software Version 20.0 (IBM Inc., Armonk, NY, USA) was used to analyze the data. Continuous data were analyzed for normality using the Shapiro–Wilks test. Differences between values were analyzed using the unpaired *t*-test (normal data), paired *t*-test (normal data), Mann–Whitney test (unpaired, non-normal data), or the Wilcoxon matched pairs test (non-normal data). A value of *p* < 0.05 indicated statistical significance.

## Results

Detailed characteristics of our cohort are shown in Table 1. There were more female than male patients (252 vs. 157). No patients had a BMI <25 kg/m^2^ (overweight), and there were 12 patients whose BMIs were >50 kg/m^2^ (super morbidly obese) at the time of their surgery. In all, 319 (78%) patients were diagnosed with T2DM preoperatively. More patients (182 vs. 227) underwent RYGB than SG.

**Figure 1:**
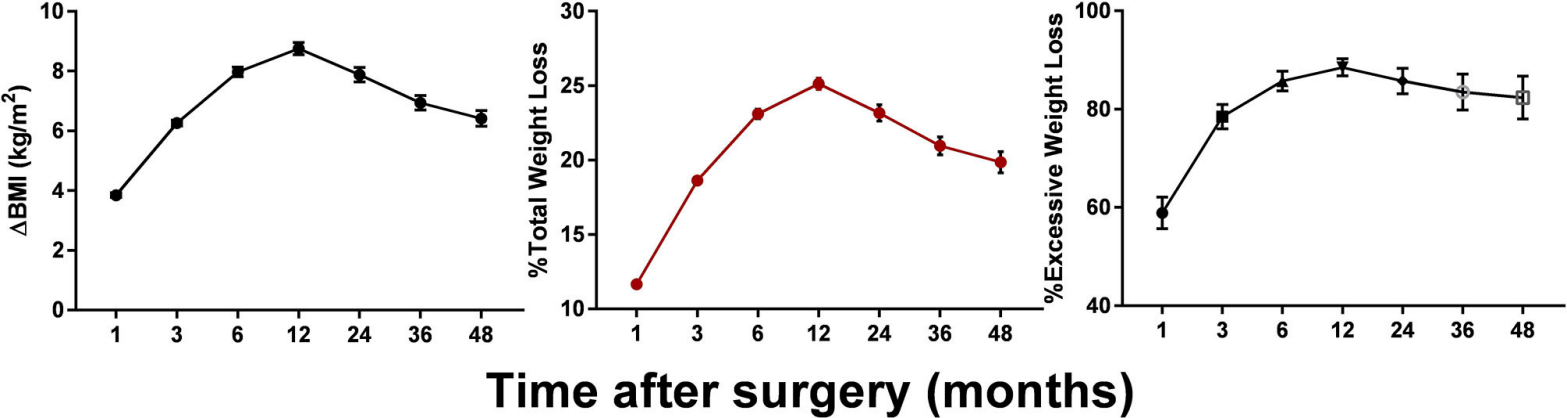
Postoperative changes of ΔBMI, %TWL, and %EWL. Results are shown in mean **±** SEM.

Postoperative changes in the BMI for up to 4 years are shown in Figure 1. The patients' BMI generally plateaued at 12 months, then decreased at a rate of 8.78 kg/m^2^. Following a slight rebound, an insignificant downward trend was observed around the mid-term, with the BMI decreasing to ~25 kg/m^2^. Weight loss of >20% TWL was achieved by 75.62, 64.66, 57.42, and 50.83% of patients at 12, 24, 36, and 48 months after surgery. Up to 53.74% of our patients lost more than 100% of their excess weight (compared with a BMI of 25 kg/m^2^) at 12 months. Although the mean weight loss stabilized at ~24 kg at 12 months, there was wide variability among the individual values, especially between the two surgical types. As can be seen from Table 2, weight loss after SG rebounded at 3 years after surgery, whereas after RYGB the weight loss was maintained.

**Table 2:** Reduction in BMI after surgery.

	**1-m post-op**	**3-m post-op**	**6-m post-op**	**1-y post-op**	**2-y post-op**	**3-y post-op**	**4-y post-op**
*n*	242	322	347	361	232	155	120
Follow-up rate	59%	79%	85%	88%	57%	38%	29%
BMI, kg/m^2^	29.07 ± 4.33	27.20 ± 4.40	26.01 ± 4.29	25.28 ± 3.88	25.40 ± 3.86*	25.68 ± 3.83*	25.58 ± 3.84*
RYGB	27.84 ± 3.18	25.59 ± 2.90	24.50 ± 3.03	24.08 ± 2.91	24.66 ± 3.34*	25.16 ± 3.34*	25.26 ± 3.64*
SG	33.30 ± 5.04	30.27 ± 5.09	28.26 ± 4.87	26.89 ± 4.42	27.38 ± 4.54*	28.46 ± 5.04	27.98 ± 4.59*
ΔBMI, kg/m^2^	3.85 ± 1.22	6.26 ± 1.78	7.97 ± 2.89	8.75 ± 3.72	7.88 ± 3.66	6.94 ± 3.07	6.42 ± 2.85
RYGB	3.65 ± 1.16	5.77 ± 1.52	6.84 ± 2.05	7.18 ± 2.24	6.81 ± 2.60	6.34 ± 2.45	6.07 ± 2.52
SG	4.53 ± 1.21	7.21 ± 1.86	9.67 ± 3.14	10.87 ± 4.22	10.75 ± 4.47	10.18 ± 4.01	8.98 ± 3.79
%TWL	11.65 ± 3.11	18.63 ± 3.92	23.11 ± 5.88	25.12 ± 7.28	23.16 ± 8.13	20.96 ± 7.41	19.86 ± 7.76
RYGB	11.55 ± 3.22	18.32 ± 4.10	21.67 ± 5.53	22.81 ± 6.15	21.47 ± 7.14	20.01 ± 7.40	19.30 ± 7.40
SG	12.00 ± 2.71	19.22 ± 3.50	25.27 ± 5.75	28.24 ± 7.54	27.72 ± 8.90	26.10 ± 8.63	24.02 ± 9.29
%EWL	58.88 ± 25.04	78.46 ± 22.31	85.71 ± 18.60	88.48 ± 16.43	85.72 ± 20.03	83.50 ± 22.85	82.33 ± 24.02
RYGB	64.15 ± 24.43	85.59 ± 18.57	91.27 ± 15.20	92.23 ± 14.28	87.64 ± 19.63	85.06 ± 22.34	83.48 ± 23.56
SG	40.83 ± 17.79	64.85 ± 22.59	77.31 ± 20.11	83.44 ± 17.79	80.55 ± 20.35	75.08 ± 24.19	73.81 ± 26.56
Percentage of follow-up patients who reached ≥50%EWL	11.98%	38.20%	49.86%	52.91%	51.29%	52.90%	50.83%
Percentage of follow-up patients who reached ≥25%TWL	0.41%	5.28%	35.16%	49.03%	39.22%	27.74%	20.83%

%TWL = $\frac{Initial\;BMI-Final\;BMI\;}{Initial\;BMI\;}$ × 100%, % EWL $=\frac{Initial\;BMI-Final\;BMI\;}{Initial\;BMI-Ideal\;BMI}$ × 100%, *P > 0.05 (vs. previous visit).

**Figure 2:**
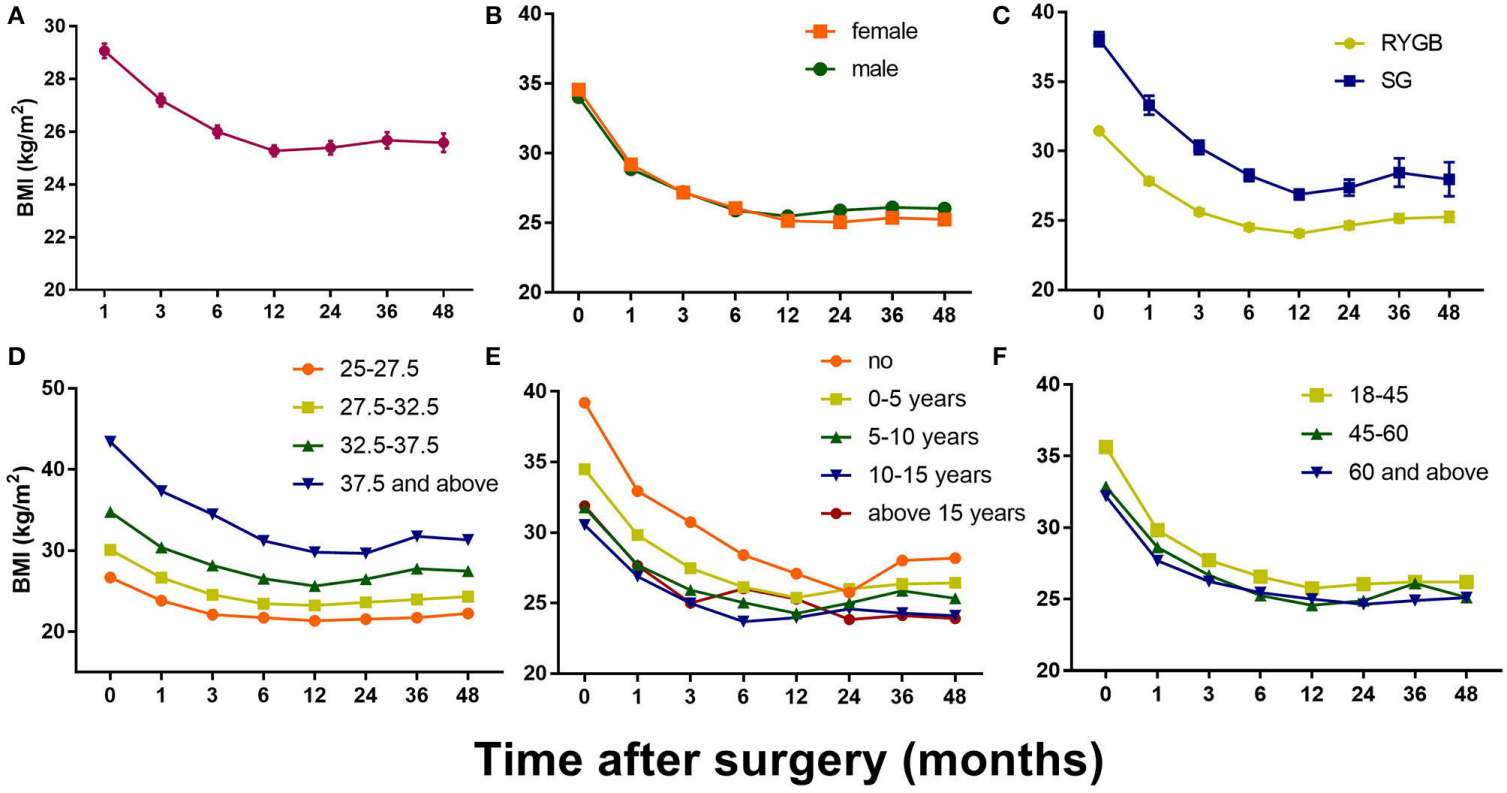
Postoperative changes of BMI in all patients and in different groups. **(A)** Mean BMI change after bariatric surgery; **(B)** Mean BMI change in different gender groups; **(C)** Mean BMI change in different surgery type groups; **(D)** Mean BMI change in different initial BMI groups; **(E)** Mean BMI change in different T2DM duration groups; **(F)** Mean BMI change in different age groups. Results are shown in mean ± SEM.

Patients were also divided into groups based on certain characteristics to explore other possibilities (Figure 2). For example, there was no significant difference between the sexes, although the female patients had generally lost more weight than the male patients at the mid-term point. In addition, post-RYGB weight loss followed a general pattern, whereas post-LSG weight loss fluctuated dramatically during the 3 years after the surgery. Divided into groups according to the preoperative BMI, patients with higher BMIs tended to lose more weight, although in a temperate slope, with the body weight dropping to its lowest 2 years postoperatively (vs. 1 year in other groups). Non-diabetic patients were more likely to have a significant weight rebound, and patients who had been diagnosed with T2DM for more than 15 years tended to face weight rebound as early as 6 months postoperatively.

The most common recipients of bariatric surgery were women of child-bearing age, accounting for 35.70% of our cohort (146 cases). Their mean BMI decreased from a baseline of 35.69 kg/m^2^ to an ideal 25.23 kg/m^2^ at 12 months and remaining at that level for 3 years postoperatively. Of these cases, we have also observed 17 pregnancies following bariatric surgery that result in different outcomes due to surgery-to-conception (S-C) interval. Two pregnancies were electively terminated during the first trimester for non-medical reasons. Five resulted in spontaneous abortions (defined as loss of pregnancy before 20 wk's gestation). The remaining 10 were carried to delivery.

## Discussion

This attempt to explore common bariatric weight loss criteria for Chinese patients showed that the body weight of bariatric surgery patients decreased sharply during the first 12–24 months, instead of at >2 years. It then stabilized unless the patient was non-diabetic, underwent SG, or had a high preoperative BMI, all of which make relapse more likely.

It is of interest to note that, the longer the patient's history of T2DM, the more likely it is that the body weight will decrease unsatisfactorily (84.7% for T2DM duration >5 years), if not rebound. This might be due to the different composition of body fat. For example, in a comparison with their BMI-matched normoglycemic counterparts, patients with T2DM have increased hepatic and pancreatic fat, which causes a severe systemic inflammatory state. A conclusive explanation for this phenomenon, however, requires further study.

Comparison of the surgical effects of the gold standard RYGB vs. the relatively new, yet predominating, SG has become a topic of debate. Our data suggest that patients who undergo SG have a significantly higher BMI than those who undergo RYGB. Both groups plateaued at 12 months, but the SG group then relapsed by a mean 3.4 kg/m^2^ 3 years later. This conclusion differs from that derived in western countries, where plateaus usually start at 18 months, probably because of the higher baseline body weight and different dietary preferences. Long-term evidence of the effectiveness of SG remains sparse, although short- to medium-term results suggest that SG may not be inferior to RYGB (14). Our results may be biased because of a larger drop-off in the follow-up of our SG patients (35.5% less than in the RYGB group). Also, because data were collected retrospectively in our study, some of the criteria selected were strongly related to whether SG was performed, including the duration of diabetes and baseline BMI. To justify the use of SG, randomized, controlled trials or cohort studies will be necessary to examine its benefit. In addition, a model that can better represent the effectiveness of bariatric surgery should be created, thereby neutralizing baseline differences that cannot be obviated.

Additionally, because most bariatric surgery is performed in women of reproductive age, defining the ideal interval between surgery and pregnancy is an emerging issue, which is partly based on the remission of obesity, which remains controversial in the present literature (15). Especially obese women in their late thirties who have failed many attempts to lose weight may look forward to a shorter interval for the good of both themselves and their children (16). Our research has indicated that women of reproductive age attain body weight stability at 12 months, which indicates a mesomeric state suitable for pregnancy. Previous studies have shown that, in obese women with polycystic ovary syndrome, hormones such as estrogen and progestogen achieve a normalized balance as early as 3 months after bariatric surgery, which has led to unplanned pregnancies (17). Therefore, guidelines for bariatric surgery often suggest that pregnancy be avoided during the first 18–24 months postoperatively—which is generally based on consensus instead of evidence (18). However, some studies have shown that there were almost no maternal or neonatal complications in women who became pregnant even within 6 months from the surgery (18, 19). In Asian settings, some research should be dedicated to determining whether the period between surgery and pregnancy can be shortened, and by how much. With our current cohort, the number of pregnancy cases was too small, thus more ought to be monitored in extended studies, in order to draw a rigorous conclusion over suggestions for conception.

The findings of this study have the potential to improve the perioperative care of patients undergoing bariatric surgery, highlighting areas for improvement in the follow-up protocol. As can be seen, although international standards (for RYGB) were followed regarding the follow-up routine, we could not identify a more precise “lowest body-weight period,” which is vital to prevent regaining the lost weight. Therefore, more frequent monthly measurements between the 6 and 12 month follow-up evaluations can be scheduled and carried further beyond the first year. With this protocol, we hope to be able to identify patients who are at high risk for treatment failure at an early stage, so help can be provided promptly. Studies have reported the importance of developing good exercise and eating habits during the early postoperative period (20). Appropriate support can also be given to patients suffering from mental health issues such as depression or anxiety (21, 22).

There are some limitations of our study. First, as a retrospective trial, there is a risk of reporting and selection bias. To lessen the impact, data were collected prospectively, and all patients who met the inclusion criteria were enrolled consecutively in the study. Second, there was a large dropout rate, especially after 2 years postoperatively. More attention should be given to long-term follow-up, including the use of specialized personnel, standardized questionnaires, and professional databases. Third, the criteria we used to divide patients into groups were not sufficiently rigid (e.g., the surgery type may be dependent on diabetes duration and the BMI). The most essential criteria will be established in future studies.

## Conclusions

Patients in a Chinese population achieved maximal weight loss and stabilization most probably between 12 and 24 months after surgery, instead of at >2 years. Further patient follow-up should include the time point of 18 month after surgery to establish more precise monthly time frames. The finding of the shorter stabilization interval is meaningful to earlier intervention of weight loss related conditions and women's conception planning.

## Data Availability Statement

The raw data supporting the conclusions of this article will be made available by the authors, without undue reservation.

## Ethics Statement

The studies involving human participants were reviewed and approved by Shanghai Jiao Tong University affiliated Sixth People's Hospital. Written informed consent to participate in this study was provided by the participants' legal guardian/next of kin.

## Author Contributions

JD and PZ conceived the presented idea. TX developed the theory and performed the computations. CW and HZ verified the analytical methods. XH and WL performed the surgery. JH and HY helped with follow-ups. JC encouraged TX to investigate the impact of pregnancy on bariatric patients. JD supervised the findings of this work. All authors discussed the results and contributed to the final manuscript.

## Conflict of Interest

The authors declare that the research was conducted in the absence of any commercial or financial relationships that could be construed as a potential conflict of interest.
